# Osteosarcoma subtypes based on platelet-related genes and tumor microenvironment characteristics

**DOI:** 10.3389/fonc.2022.941724

**Published:** 2022-09-23

**Authors:** Yuan Shu, Jie Peng, Zuxi Feng, Kaibo Hu, Ting Li, Peijun Zhu, Tao Cheng, Liang Hao

**Affiliations:** ^1^ Departments of Orthopedics, Second Affiliated Hospital of Nanchang University, Nanchang, China; ^2^ The Second Clinical Medical College of Nanchang University, Nanchang, China; ^3^ Department of Orthopaedic Surgery, Shanghai Jiao Tong University Affiliated Shanghai Sixth People’s Hospital, Shanghai, China

**Keywords:** platelet, cancer, immunology, prognosis, subtypes, microenvironment

## Abstract

**Background:**

Osteosarcoma is a common metastatic tumor in children and adolescents. Because of its easy metastasis, patients often show a poor prognosis. Recently, researchers have found that platelets are closely related to metastasis of a variety of malignant tumors, but the role of platelets related characteristics in osteosarcoma is still unknown. The purpose of this study is to explore the characteristics of platelet-related subtypes and cell infiltration in tumor microenvironment.

**Methods:**

We collected osteosarcoma cohorts from TCGA and GEO databases, and explored the molecular subtypes mediated by platelet-related genes and the related TME cell infiltration according to the expression of platelet-related genes in osteosarcoma. In addition, we also explored the differentially expressed genes (DEGs) among different molecular subtypes and established a protein-protein interaction network (PPI). Then we constructed a platelet scoring model by Univariate cox regression and least absolute shrinkage and selection operator (Lasso) cox regression model to quantify the characteristics of platelet in a single tumor. RT-PCR was used to investigate the expression of six candidate genes in osteosarcoma cell lines and normal osteoblast lines. Finally, we also predicted potential drugs with therapeutic effects on platelet-related subtypes.

**Results:**

We found that platelet-related genes (PRGs) can distinguish osteosarcoma into two different platelet-related subtypes, C1 and C2. And the prognosis of the C2 subtype was significantly worse than that of C1 subtype. The results of ESTIMATE analysis and GO/KEGG enrichment showed that the differences between different subtypes were mainly concentrated in immune response pathways, and the immune response of C2 was inhibited relative to C1. We further studied the relationship between platelet-related subtypes and immune cell infiltration. We found that the distribution of most immune cells in C1 subtype was higher than that in C2 subtype, and there was a correlation between C1 subtype and more immune cells. Finally, we screened the PRGs related to the prognosis of osteosarcoma through Univariate Cox regression, established independent prognostic platelet characteristics consisting of six genes to predict the prognosis of patients with OS, and predicted the drugs that may be used in the treatment of osteosarcoma. RT-PCR was used to verify the expression of candidate genes in osteosarcoma cells.

**Conclusion:**

Platelet scoring model is a significant biomarker, which is of great significance to determine the prognosis, molecular subtypes, characteristics of TME cell infiltration and therapy in patients with OS.

## Introduction

Osteosarcoma (OS) is the most common bone cancer among children and adolescents (1), accounting for nearly two-thirds of all primary bone malignancies (2, 3). OS, characterized by poor prognosis and a high disability rate (4), is mainly caused by metastasis, particularly lung metastasis. According to follow-up data, the five-year survival rate of patients with primary OS reduces from 75% to 35% once lung metastasis occurs (5–7). Although advances in the treatment of OS have significantly improved the survival rate of these patients (8), the complexity and instability of the genome exert a significant impact on treatment outcomes (9, 10). Therefore, early diagnosis, treatment, and prognosis of OS need to be optimized from the perspective of molecular genetics.

As an important part of osteosarcoma microenvironment, platelets participate in the growth and metastasis of osteosarcoma and have great potential in osteosarcoma targeted therapy (11). More than 30% of the patients with malignant solid tumors also show simultaneous thrombocytosis, which decreases their survival rates (11, 12). This phenomenon occurs as tumor-derived IL-6 stimulates an increase in thrombopoietin levels, resulting in thrombocytosis (13). Increased platelet activity can promote tumor growth and metastasis and reduce the effectiveness of immunotherapy on tumor cells (14). Tumor cells induce platelet aggregation, and platelets wrap tumor cells in the thrombus to prevent them from being attacked by natural killer (NK) cells, thereby reducing the tumor cell surveillance by immunogenic cells (15, 16). Platelets can also help tumor cells enter the circulatory system, and adhere to the endothelium, resulting in their metastases (17). Moreover, platelets can produce TGF-β, which not only inhibits IFN-γ and decreases the cytotoxicity of NK cells but also increases the tumor cell metastases (18).

However, as an important part of tumor microenvironment, the role of platelets in osteosarcoma (OS) has not been fully studied. Therefore, we identified two different platelet subtypes in OS through TCGA and GEO databases, and systematically analyzed the expression of PRGs and their impact on patients’ prognoses and TME. Next, we evaluated the molecular characteristics, prognostic significance, and abundances of infiltrating immune cells in different subtypes. Additionally, we predicted the potential drugs for the treatment of OS based on the DEGs between different groups divided by platelet scores, and finally verified the expression of six candidate genes between OS and normal cell lines through *in vitro* experiments. Our findings will help expand the knowledge of the role of platelets in OS and facilitate the development of new therapeutic interventions.

## Methodology

### Data acquisition

The sample data were screened from TCGA and GEO databases. The inclusion criteria were as follows: (a) determined as an OS sample; (b) availability of survival status and corresponding survival time, and (c) expression of more than half of the genes. We screened 85 samples from the Target database, which comprised the training set, and 53 from the GSE21257 dataset in the GEO database, comprising the verification set. The list of platelet-related genes (PRGs) was obtained from the MSigDB gene set (https://www.gsea-msigdb.org) using the keyword “platelet”. We finally obtained 480 PRGs for further analyses.

### Unsupervised clustering of PRGs

First, the expression profiles of 480 PRGs in patients with OS were extracted and 112 prognostic PRGs were obtained using Univariate Cox regression. According to the levels of expression of these 112 genes, 85 samples in TCGA were classified by unsupervised clustering for further analyses. The consensus clustering (CC) algorithm was used to determine the number of clusters to identify unrecognized subtypes in OS (19), and the “ConsensusClusterPlus” package was also used. “MaxK” was selected as 10, and “Pearson” was used for the “clustering method” and “KM” for the “cluster distance”. The Stromal, Immune, and Estimate scores, along with tumor purity in malignant tissues were obtained using the ESTIMATE algorithm.

### Functional and pathway enrichment analyses

Using the R software, the enrichment score for each sample in the gene set from GSVA was computed (20), and “c2.cp.kegg.v7.4.symbols.gmt” was extracted from MSigDB to evaluate relevant pathways and molecular mechanisms. The minimum and the maximum gene sets were set from 5 to 5000, respectively. We calculated the enrichment score for each sample in the gene set and obtained the enrichment matrix. Different pathways were identified between the two clusters using the “limma” package.

### Assessment of the tumor microenvironment (TME) in OS

From previous studies, 28 types of immune cell-related genes were identified (21), and the corresponding immune cell infiltration based on the expression of these genes was predicted for the samples by ssGSEA. Thus, the infiltration profile of these immune cells was obtained.

### Differentially expressed genes (DEGs) between platelet subtypes

DEGs between different platelet subtypes were analyzed using the “limma” package. In order to increase the accuracy, the following screening conditions were set: |logFC| > 2, p < 0.05. Then, a protein-protein interaction network (PPI) of DEGs was constructed using STRING and visualized on Cytoscape. Kyoto Encyclopedia of Genes and Genomes (KEGG) and Gene Ontology (GO) enrichment analyses were conducted for the enrichment analysis of DEGs. ClueGO, a plug-in of Cytoscape, was also used to conduct the enrichment of these DEGs.

### Construction and validation of the prognostic model based on PRGs

Univariate Cox regression was used to screen prognostic PRGs in the GSE21257. To narrow the screening range, commonly shared genes between training and validation sets were obtained using a Venn diagram. And eight common PRGs were obtained. LASSO-Cox regression analysis further reduced the preliminary screening range of prognostic PRGs. The prognostic model was constructed as follows: risk score = ∑ in (CoefixXi), where X represents the level of expression of each PRG and CoefixXi represents the coefficient of relative levels of prognostic PRGs based on the LASSO regression model. The risk score for each sample was evaluated using a prognostic model. The R package, “maxstat” (maximally selected rank statistics with severe p-value approximations version: 0.7-25), was used to calculate the optimal cut-off for the risk score; set the minimum and maximum numbers of grouped samples to >25% and <75%, respectively. We then obtained the optimal cut-off and used it as the critical value for the high- and low-risk groups. Those patients having a score above the critical were included in the high-risk group, while the remaining were included in the low-risk group. Furthermore, R package, “survival”, was used to analyze the prognostic differences between the groups, and the significance of these differences was evaluated using a log-rank test. Additionally, the GSE21257 dataset from GEO was the validation set used to verify the predictive effect of our prognostic model.

### Real-time RT-PCR

Total RNA was extracted from various OS cells and the normal cell line, hfoB1.19, using TRIzol (Invitrogen) reagent, and reverse-transcribed to synthesize the cDNA using the Prime Script TMRT kit (Takara, RR047A). The SYBR Premix Ex in the Taq II Kit was used and PCR amplification was performed following the manufacturer’s instructions. The primer sequences used were listed in **Table 1**.

**Table 1:** The Primer pairs utilized in Real-Time PCR.

Genes	Primer- Forward (5’-3’)	Primer-Reverse (5’-3’)
TGFB2	GAGTGCCTGAACAACGGATT	CCATTCGCCTTCTGCTCTTG
GNG12	ACAATATAGCCCAGGCAAGGAG	CACTCCTGGCATGTTCCTCAC
KIF21B	GGTGTCATCAAGGTCTGGAAC	CTGGAGGCTGTGAAGATATGC
ANXA5	CAAGCCTGGAAGATGACGTG	TCAATTCCAGCTCAGGGTCT
GAS6	TGGCATGTGGCAGACAATCT	ATACCTCCCACGGTCAGGTT
MAPK1	GGCTGTTCCCAAATGCTGAC	AACTTGAATGGTGCTTCGGC
GAPDH	ACAACTTTGGTATCGTGGAAGG	GCCATCACGCCACAGTTTC

### DEGs in the risk groups and drug prediction

The DEGs between the high- and low-score groups were screened using the “limma” package, and we selected the top 50 upregulated and downregulated genes showing the highest significant difference. Drugs interacting with DEGs were predicted using DGIdb (https://dgidb.org), a database of information on the association of genes with their known or potential drugs. The screening conditions were set as follows: (a) clear interaction between genes and drugs and (b) drugs published previously in the literature.

### Statistical analysis

All statistical analyzes were performed using R 4.0.5 (https://www.r-project.org/). The Wilcoxon rank-sum test was used to verify the results of the ESTIMATE analysis. Kaplan–Meier curves were used to compare the differences in survival rates between the risk groups, and Univariate Cox regression analysis was performed to screen the relevant genes. Statistical significance was set at p < 0.05. *p < 0.05, **p < 0.01, ***p < 0.001.

## Results

### Identification of platelet subtypes in OS

**Figure 1:**
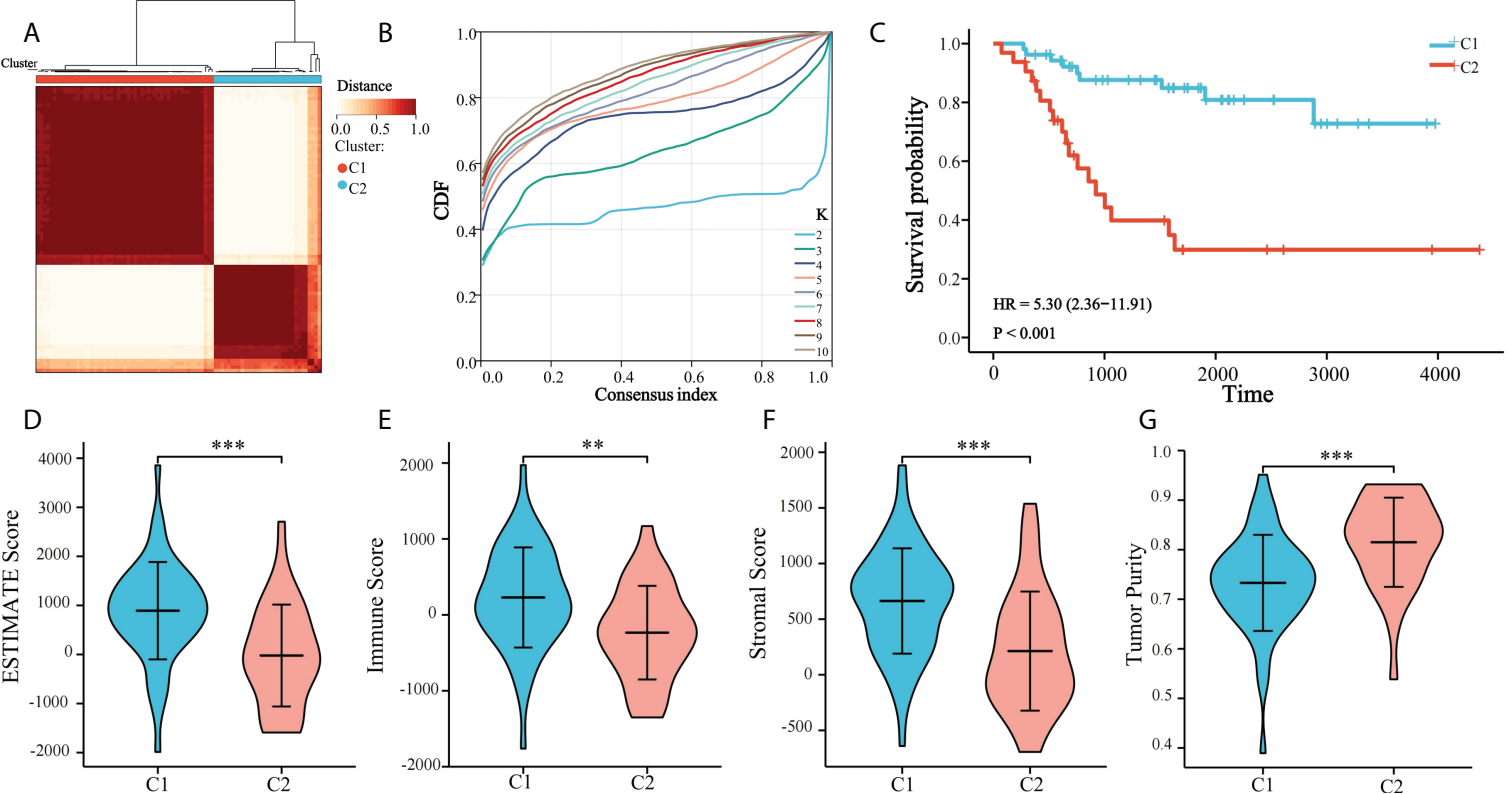
Unsupervised clustering of PRGs. **(A)** Clustering heatmap of prognosis-related platelet genes, 85 samples of TCGA were divided into 2 groups (K = 2); **(B)** Consensus Cumulative Distribution Function (CDF) plot under k = 2–10, where the number of k represents the number of groups after unsupervised clustering; and **(C)** Survival of patients in C1 and C2; **(D–G)** ESTIMATE Score, Immune Score, Stromal Score, Tumor Purity of C1 and C2 (**p < 0.01, ***p < 0.001).

Platelet-related subtypes and platelet characteristics were analyzed. TCGA cohort consisted of 85 patients, and their survival and clinical conditions were recorded. Univariate Cox regression analysis was used to screen for 112 prognostic PRGs in TCGA (**Supplementary Figure 1**), whereby their expressions in 85 OS samples were analyzed by unsupervised clustering (**Figure 1A**). The results suggested that at k = 2, the differences between subgroups were significant, indicating that the 85 patients with OS could be divided into two groups, namely C1 and C2 (**Figure 1B**). C1 and C2 comprised 53 and 32 cases, respectively. Survival analysis showed that the prognoses were significantly different between the two platelet subtypes. C1 showed a clear survival advantage, whereas patients in C2 had relatively poor survival (**Figure 1C**). Additionally, the ESTIMATE algorithm was used to estimate the TME of the samples and comparisons were made between the subtypes. OS samples in C1 had higher stromal, immune, and estimated scores, and lower tumor purity relative to those in C2 (**Figures 1D–G**). This further explained the phenomenon that the survival condition of C2 is worse than that of C1.

### Comprehensive analysis of platelet DEGs in OS

**Figure 2:**
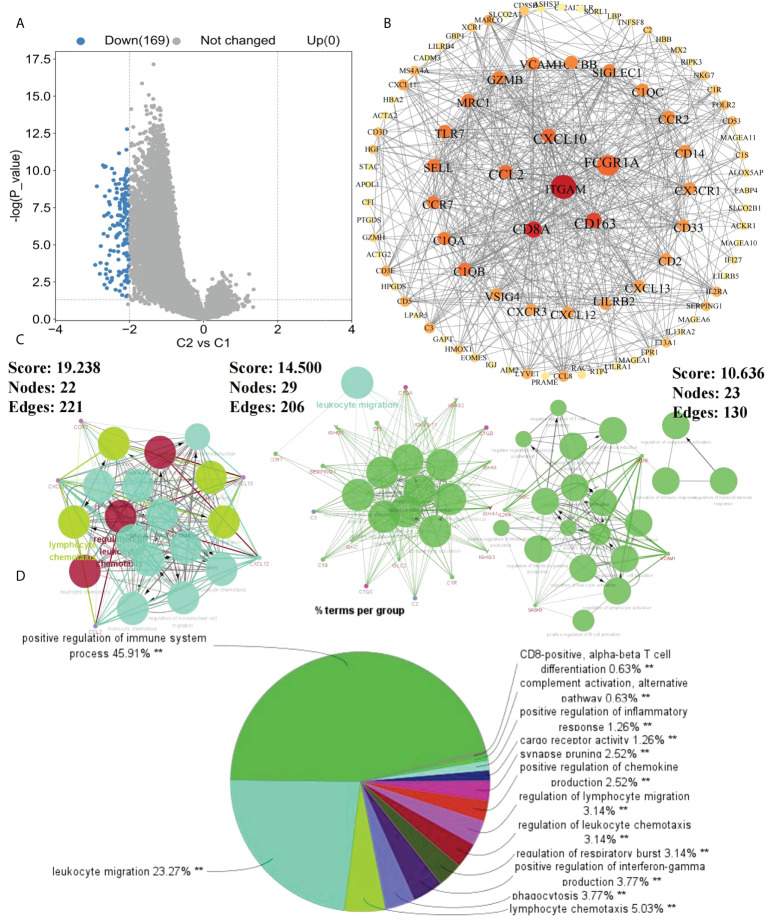
Enrichment results of DEGs. **(A)** DEGs between C1 and C2 subtypes (p<0.05, |logFC>2|); **(B)** Protein-Protein interaction (PPI) network of 169 DEGs between C1 and C2 subtypes; **(C)** Key modules in PPI selected according to MCODE; **(D)** ClueGo enrichment results of DEGs (**p < 0.01).

**Figure 3:**
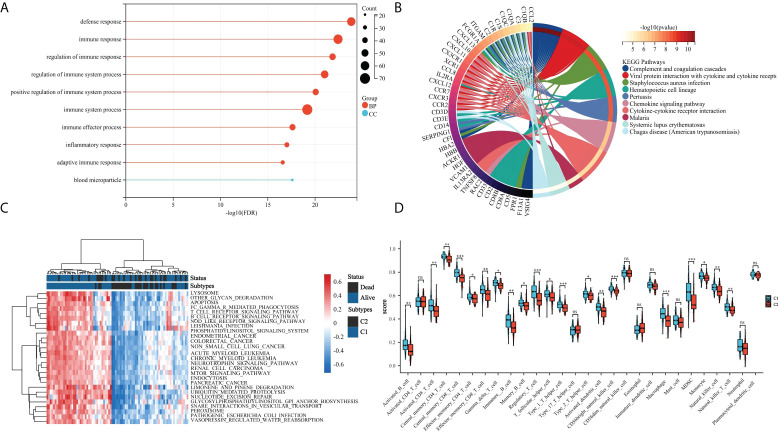
Assessment of the tumor microenvironment in patients with OS. **(A)** Differences in immune cell infiltration between C1 and C2 subtypes; **(B)** Enrichment heatmap of GSVA analysis, navy blue indicates C1 and dark color means C2; **(C)** Gene Ontology (GO) enrichment results of DEGs; and **(D)** Kyoto Encyclopedia of Genes and Genomes (KEGG) pathway enrichment analysis of DEGs. *p < 0.05,**p < 0.01, ***p < 0.001 ns means no significant difference.

To further investigate the effects of subtypes on patients, DEGs between C2 and C1 were screened. With “|logFC| > 2 and p < 0.05” as the screening condition, 169 DEGs were obtained, all of which were downregulated in C2 compared with in C1 (**Figure 2A**). Using STRING and Cytoscape, a PPI network was constructed for these DEGs and using ClueGO, enrichment analysis was performed (**Figure 2B**). Finally, we extracted three key modules and visualized all enrichment results (**Figures 2C, D**). Additionally, GO and KEGG analyzes were performed to assess the enrichment features for the 169 DEGs. Blood microparticles, defense responses, immune responses, immune system processes, positive regulation of immune system processes, and other biological functions related to immunity were significantly enriched in GO analysis (**Figure 3A**). KEGG analysis revealed that the DEGs were significantly enriched in complement and coagulation cascades, hematopoietic cell lineage, and chemokine signaling pathway (**Figure 3B**). Since the expression of DEGs in C2 was lower than that in C1, these pathways were more likely inhibited in C2. The inhibition of blood microparticles and complement and coagulation cascades probably implied that the synthesis and release of platelets and the activation processes in the C2 subtype were inhibited (22, 23). Thus, we thought that patients with the C2 subtype have lower platelet content or lowered function than those with subtype C1.

These results also showed that the immune function in C2 was inhibited as compared to that in C1. Platelets are involved in immune processed and are significantly related to immune responses (24, 25). Therefore, we reasonably speculated that the patient’s immune status represented the level of platelets to a certain extent. Therefore, the subtypes according to PRGs could significantly distinguish the status of platelets in patients, and this feature could be expressed through their immune statuses.

### Immune cell infiltration between platelet subtypes

**Figure 4:**
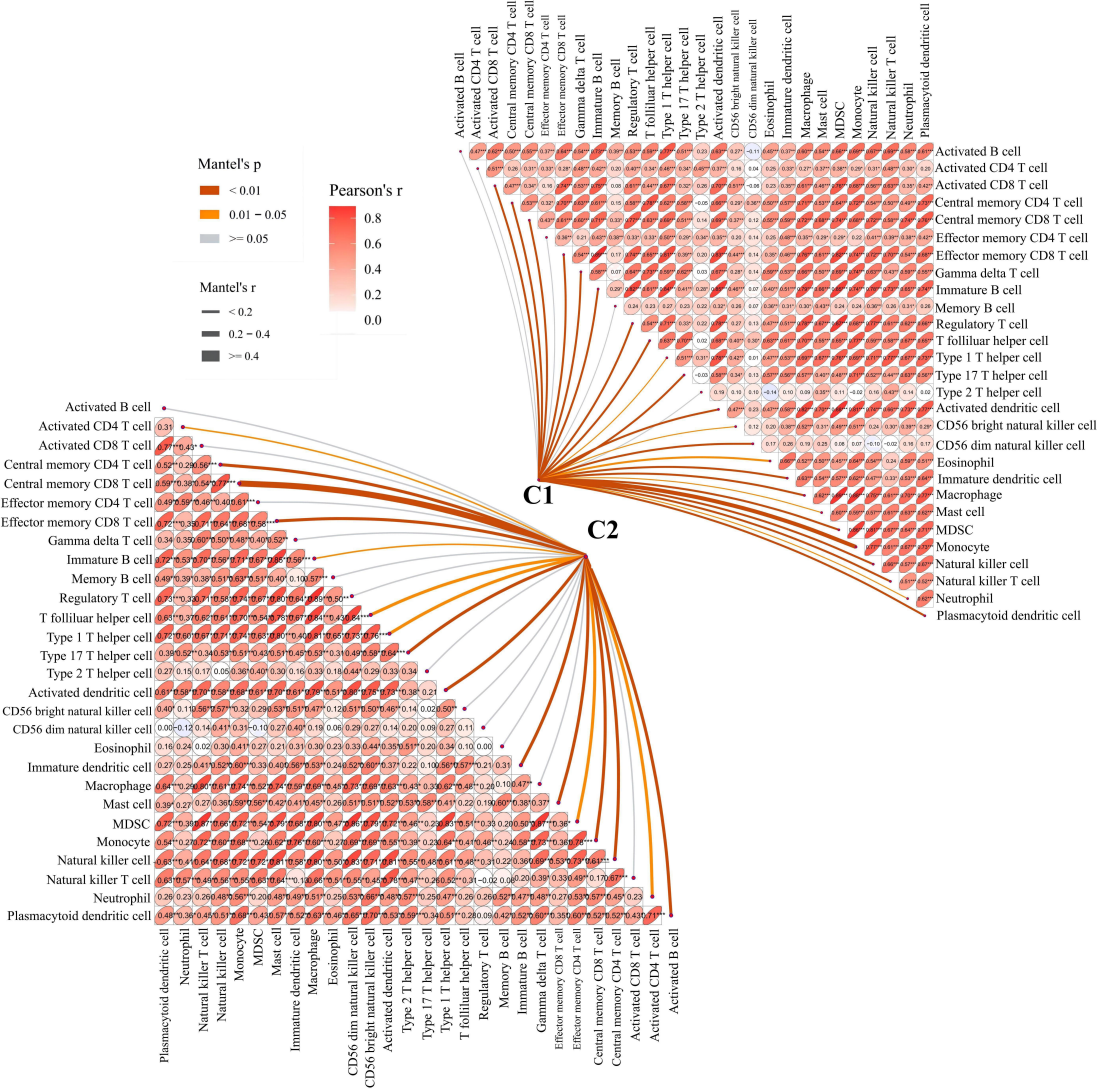
Correlation between C1 and C2 groups and 28 immune cells. The line width represents the r value of mentel; the line color represents the p value of mentel analysis results; the numbers in the ellipse represent Pearson’s coefficients.

To further examine the relationship between platelets and immunity, GSVA was performed and different pathways were enriched between subtypes. For example, as compared to C1 with a clear prognostic advantage, B-cell receptor, T-cell receptor, and nod-like receptor signaling pathways were significantly downregulated in C2 (**Figure 3C**). These pathways are highly correlated with immune functions, indicating that the immune function in C2 was inhibited relative to C1. The infiltration in TME between the platelet subtypes was analyzed using ssGSEA (**Figure 3D**). Among the 28 immune cells, 20 were significantly enriched in C1, including activated B cells, activated CD8 T cells, central memory CD4 T cells, effector memory CD8 T cells, and gamma delta T cells. This phenomenon verified our conjecture. Further studies on the correlation between tumor subtypes and immune cells showed greater abundance correlated significantly with C1. Activated CD8 T cell, Gamma delta T cell, Regulatory T cell, CD56 bright natural killer cell, CD56 dim natural killer cell, Eosinophil, Macrophage and Natural killer T cell were only significantly related to C1 (**Figure 4**). These immune cells play a crucial role in anti-tumor effect and leading to a good prognosis for patients (26–29).

Combined with the above findings, we believe that the immune function in C2 was indeed inhibited relative to C1. This is consistent with our previous conclusion, whereby, the platelet content or function of patients with the C2 subtype was lower than those with the C1 subtype, which was marked by an inhibited immune status relative to the patients with the C1 subtype.

### Construction of model based on platelet characteristics

**Figure 5:**
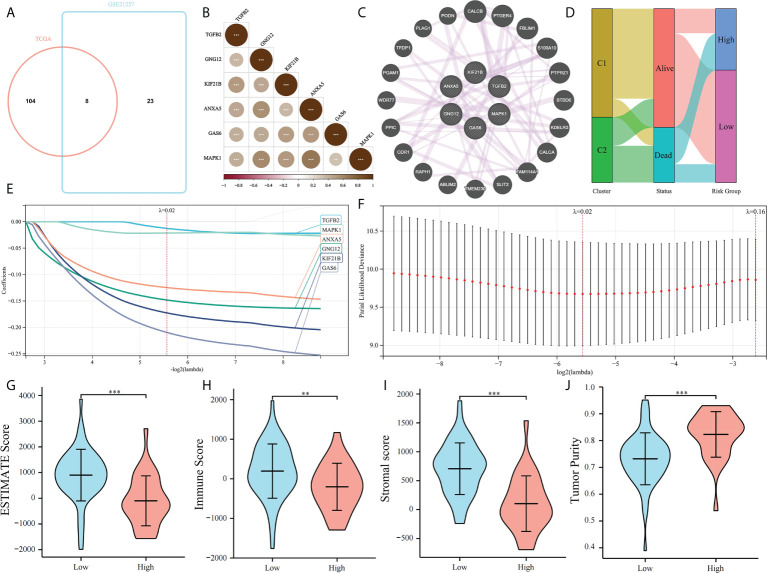
Construction of platelet characteristic score model. **(A)** Venn map screening of platelet related genes associated with prognosis in both TCGA and GSE21257 cohorts; **(B, C)** Platelet characteristic score model composed of six candidate genes screened using the LASSO analysis with minimal lambda; **(D)** Heatmap of correlation among six candidate genes; **(E)** The co-expression relationship of six candidate genes explored by Genemania; **(F)** Sanggi diagram showing the relationship between subtype, survival status, and platelet score; and **(G–J)** ESTIMATE Score, Immune Score, Stromal Score, and Tumor Purity of a high-platelet group and low-platelet score groups. **p < 0.01, ***p < 0.001.

The prognostic value of PRGs was examined. To improve the accuracy of our results, Univariate Cox regression was performed to screen prognostic PRGs in the validation set GSE21257 (**Supplementary Table 1**). The prognostic PRGs from the two cohorts were intersected, eight common prognostic PRGs were screened (**Figure 5A**). LASSO Cox regression was performed to further identify prognostic genes and six genes were selected, MAPK1, GNG12, KIF21B, ANXA5, GAS6, and TGFB2. We found that MAPK1, GNG12, ANXA5, GAS6, and TGFB2 have been reported to be involved in platelet activation and function regulation (30, 31). For example, MAPK1 is related to the activation pathway of platelets (32) and ANXA5 can inhibit the production of thrombin and participate in the regulation of platelet aggregation (33). However, the relationship between KIF21B and platelets has not been studied. Then we establish a platelet prognostic model based on the expression of these six common prognostic genes in TCGA (**Figures 5E, F**), they were used to define the platelet score. We also analyzed the correlations among the six genes and found a significant co-expression relationship (**Figures 5B, C**). According to the best intercept value, 85 patients with OS were divided into the high- (n = 30) and low- (n = 55) platelet score groups. The relationship between platelet score and survival status with platelet groups was assessed, and the results were consistent with our expectations. The survival of patients in C1 was significantly better than those in C2, and most of them had a low platelet score (**Figure 5D**). This validated the effectiveness of our six gene model. The ESTIMATE results showed that in the low platelet score group, the ESTIMATE, immune, and stromal scores were significantly higher; tumor purity was higher in the cohort with high platelet scores (**Figures 5G–J**). This indicated that in the cohort with a low platelet characteristic score, the infiltration of immune and stromal cells was higher, consistent with our previous conjecture that platelet characteristics were related to the TME.

**Figure 6:**
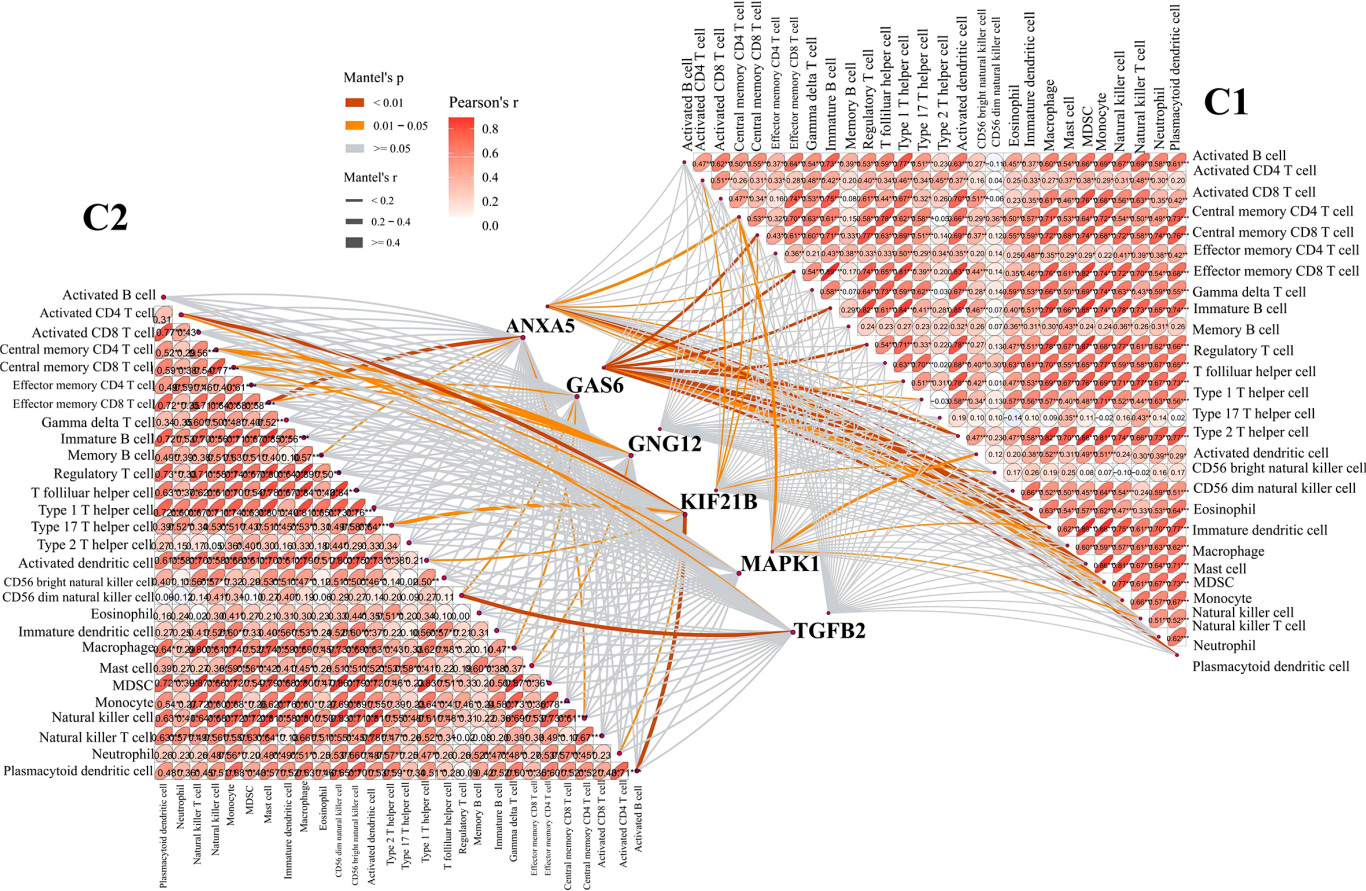
Correlation between six candidate genes and 28 immune cells. The line width represents the r value of mentel; The line color represents the p value of mentel analysis results; the numbers in the ellipse represent Pearson’s coefficients.

Moreover, the relationship between these six genes and immune cells was assessed. We found that most of the six candidate genes showed correlation with immune cells, and GAS6 showed the strongest correlation with immune cells in C1 subtype (**Figure 6**). This indicates that GAS6 may play a great role in the immune difference between C1 and C2 subtypes.

### Verification of the model based on platelet characteristics

**Figure 7:**
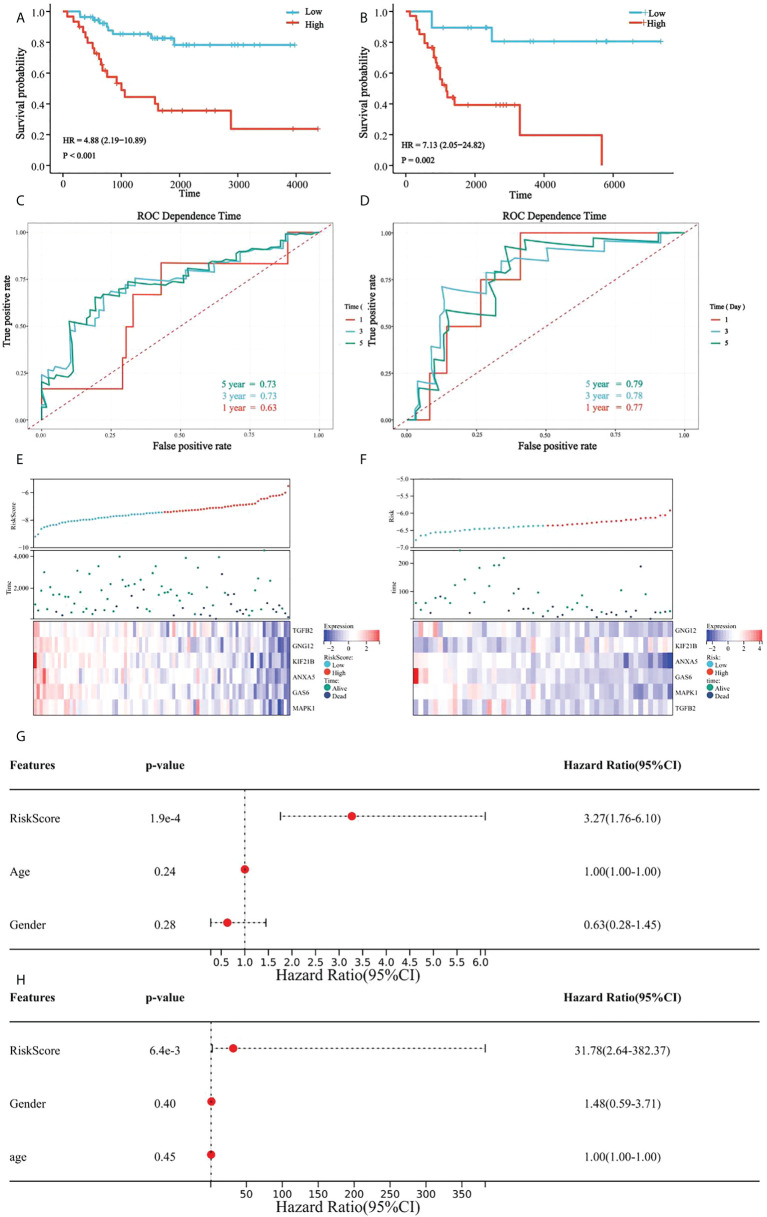
Verification of platelet score characteristics. **(A)** Survival difference between high-rating and low-rating groups in the TCGA cohort; **(B)** Survival difference between high-rating and low-rating groups in the GSE21257 cohort; **(C)** Time-dependent ROC analysis of TCGA cohort; **(D)** Time-dependent ROC analysis of the GSE21257 cohort; **(E)** Candidate gene expression and survival heatmap in TCGA cohort; **(F)** Candidate genes expression and survival heatmap in the GSE21257 cohort; **(G)** Forest diagram of multivariate results of platelet characteristic score model and clinical factors in TCGA cohort; and **(H)** Forest diagram of multivariate results of platelet characteristic score model and clinical factors in the GSE21257 cohort.

In all patients, a high platelet score was associated with a lower survival rate, and the Kaplan-Meier curve indicated significant differences between the groups (**Figure 7A**). Our prognostic model was further evaluated using time-dependent ROC analysis. The area under the ROC curve (AUC) was 0.63, 0.74, and 0.72 for 1-, 3-, and 5 years, respectively (**Figure 7C**). Moreover, the heatmap showed that the expression of the six candidate genes correlated negatively with patient survival (**Figure 7E**).

Similarly, the results in the validation set, GSE21257, suggested significant differences in survival between the groups with high- and low platelet scores (**Figure 7B**). The AUC was 0.78 for 1-year, 0.79 for 3-year, and 0.80 for 5-years (**Figure 7D**). Heatmap also showed that the expression of the six candidate genes correlated negatively with patient survival (**Figure.7F**).

Multivariate Cox regression analysis was used to determine the relationship between the platelet score and clinical features. We found that the platelet score model was an independent prognostic factor (**Figure 7G**). Similar results were obtained in GSE21257 (**Figure 7H**). These results suggested that platelet characteristics were independent prognostic factors for OS and had a good power to effectively predict the prognosis of OS patients.

### Expression of six genes in different OS cell lines

**Figure 8:**
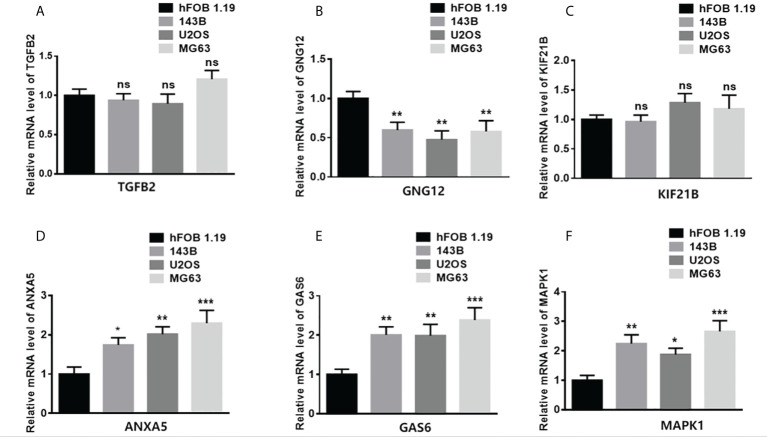
Validation of gene expression in normal and tumor samples. **(A–F)** represent ANXA5, GAS6, GNG12, KIF21B, MAPK1, and TGFB2 respectively. (*p < 0.05,**p < 0.01, ***p < 0.001 ns means no significant difference).

To study the gene expression patterns in tumor versus normal cell lines, total RNA from different tumor cell lines (143B, U2OS, and MG63) and a normal osteoblast cell line (hFOB 1.19) was extracted. The levels of mRNA expression of TGFB2, GNG12, KIF21B, ANXA5, GAS6, and MAPK1 were assessed (**Figure 8**). The expression of ANXA5, GAS6, and MAPK1 in the tumor cells was significantly higher than that in the normal cell line. However, the expression of GNG12 in the tumor cells was significantly lower than that in the normal cell line.

### Drug prediction based on platelet characteristic DEGs

To examine the clinical effects of platelet characteristics on OS treatment, DEGs between the high and low platelet score groups in the TCGA cohort were screened. To prevent the omission of important genes, our screening condition was set at “|logFC| > 1 and p <0.05”. The top 50 most significantly upregulated and downregulated genes were selected, respectively. And these 100 genes were uploaded to the DGIdb database (https://dgidb.org) for drug prediction. The drug screening conditions were as follows: (a) clear relationship with the target gene and (b) the effects of the drug on target genes reported previously in the literature. Finally, 15 drugs with the top scores were obtained (**Table 2**).

**Table 2:** Information of the top 15 drugs filtered by Interaction score.

Gene	Drug	Interaction_types	Interaction score	PubMed ID
SOST	ROMOSOZUMAB	inhibitor	63.65	28755782
PTGFR	TRAVOPROST	agonist	48.67	19929706|18983226
PTGFR	LATANOPROST	agonist	19.97	15037111|9733584
PTGFR	TAFLUPROST	agonist	14.98	21858491
PTGFR	BIMATOPROST	agonist	14.98	14733708|17724194
SLC6A2	REBOXETINE	inhibitor	9.09	12388649
SLC6A2	DEXMETHYLPHENIDATE	inhibitor	7.14	11160413|18480678
SLC6A2	GUANETHIDINE	inducer	6.5	16126010|8710929
CA3	ACETAZOLAMIDE	inhibitor	4.9	20605094|1909176
SLC6A2	ATOMOXETINE	inhibitor	4.42	14709944|16142049
SLC6A2	PHENMETRAZINE	inhibitor	3.9	12106802|17139284
PTGFR	DINOPROST TROMETHAMINE	agonist	3.74	8777582
CA3	ETHOXZOLAMIDE	inhibitor	3.26	17826101
SLC6A2	DIETHYLPROPION	inhibitor	3.25	19897080|17139284
SLC6A2	SIBUTRAMINE	inhibitor	3.03	16678551|19475780

## Discussion

Chemotherapy has shown progress in the treatment of OS in recent years. However, drug resistance in tumor cells is inevitable during chemotherapy (34). Previous studies have also identified distinct molecular subtypes of OS. However, significant heterogeneity and limitations remain (35, 36). Therefore, more accurate typing of OS is required for targeted treatment and to improve the survival rate of these patients. The effects of platelets on osteosarcoma are diverse and complex. For example, osteosarcoma cells usually show high platelet activation inducing characteristics, which can induce platelet activation. Activated platelets secrete LPA and CLEC, which enhance the invasive ability of osteosarcoma through the LPA-LPAR1 axis and the interaction between platelet CLEC-2 and osteosarcoma podoplanin (37, 38). And tumor cells can release soluble mediators such as ADP (16) to activate platelets and form polymers through TCIPA on the surface of tumor cells, so that platelets can wrap tumor cells, thus avoiding the attack of immune cells in the circulatory system (39). Therefore, platelet-cancer cell interaction and activated platelets releasing bioactive molecules are very important for hematogenous metastasis of osteosarcoma. Moreover, there are a large number of growth factors stored in the Alpha (α) granules of platelets. When platelets contact with osteosarcoma cells, they secrete a variety of growth factors, such as TGF- β, and VEGF, which can induce osteosarcoma cells to express tissue factor and promote tumor growth (40–42). But another thing worth noting is that platelets also have an anti-tumor effect. Platelets can inhibit tumor growth by transporting mir-24 into cancer cells targeting mt-Nd2 and Snora75 (43). This indicates that the regulatory effect of platelets on tumor growth is multiple. Additionally, platelets also have a great impact on the treatment of patients with osteosarcoma. For example, platelet-derived growth factor recepter can be used as a target for imatinib, mesylate, and other inhibitors in the treatment of osteosarcoma (44, 45).

Therefore, the study of platelet characteristics may provide new targets for cancer therapy (46–48). However, the relationship between OS and platelets remains unclear. In our study, we examined the expression of PRGs in OS and the comprehensive role of the TME. We found that OS could be divided into two subtypes based on the expression of 112 PRGs. The prognostic differences, stromal, estimate and immune scores and tumor purity between the subtypes were studied, as were the differences between pathway enrichment and DEGs. The results showed differences in the abundance and activity of platelets among different subtypes, evidenced by differences in immune statuses and prognoses of patients. GSVA showed immune-related pathways were significantly downregulated in C2. According to previous studies, platelets are closely associated with cancer immunity (49). These can act against apoptosis through proliferation signals, and angiogenic factors can enhance tumor growth (50). Therefore, two platelet subtypes and the TME cell infiltration were assessed. The degree of infiltration of most immune cells in C2 was significantly lower than that in C1, consistent with our previous conclusion that C2 showed higher immunosuppression as compared to C1. Further analysis of the relationship between immune cells and subtypes showed that activated CD8 T cell, gamma delta T cell, regulatory T cell, CD56 bright natural killer cell, CD56 dim natural killer cell, eosinophil, and macrophage only correlated significantly with C1. These immune cells are particularly important for immune function, especially CD56 bright natural killer cell and CD56 dim natural killer cell (26). By exploring the relationship between candidate genes and immune cells, we found that GAS6 showed a strong correlation with immunity and cells in C1, which was not reflected in C2 subtype. By working with Alx, GAS6 can promote immunosuppressive TME and participate in the recruitment of specific immune cells (50). This may be one of the reasons for the immune difference between C1 subtype and C2 subtype. However, a more accurate explanation requires further investigation.

Considering the impact of the platelet score on the prognosis of OS, a blood platelet model based on the six key platelet genes was constructed, MAPK1, GNG12, KIF21B, ANXA5, GAS6, and TGFB2. The growth arrest specific gene 6 product (GAS6) is an anticoagulant protein related protein that can enhance platelet aggregation and secretion, thereby enhancing platelet response (30). Carthamus tinctorius L is a drug widely used in cardiovascular therapy. As a core gene of platelet activation pathway, mapk1 can be regulated by Carthamus tinctorius L to inhibit platelet aggregation (32). Phosphotidylserine (PS) participates in the activation process of platelets and can regulate the production rate of thrombin in plasma, while Annexin A5 (ANXA5) can interact with PS to reduce the concentration of PS and then inhibit the production rate of thrombin (33). Central regulating signaling cascade of platelets (CC) mainly regulates the interaction between exogenous factors and platelets. GNG12 can act on CC and regulate platelet production (51). Platelet characteristics were used to quantify the platelet scores. As expected, the platelet score in C2 was higher than that in C1. The prognosis of patients with low platelet scores was also better than those with high platelet scores. These results were verified in GSE21257. Platelet scores can thus be used as an independent prognostic factor for patients with OS.

As far as we know, this is the first bioinformatic study that reveals the platelet-related characteristics in OS. We identified platelet-associated subtypes through platelet characteristic gene pairs and explored the differences in gene expression, TME, and prognosis among these subtypes. We believe that the characteristic genes not only can be used as a prognostic biomarker in clinical settings but also provide a new direction for the treatment of OS. Moreover, we also predicted potential therapeutic drugs for the treatment of OS.

## Conclusion

Overall, we defined two molecular subtypes with different prognoses based on PRGs in OS. The platelet scoring model is a significant biomarker for identifying the prognosis, molecular subtypes, characteristics of TME cell infiltration, and treatment of patients with OS. But the relationship between platelets and immunity at the cellular and molecular levels needs to be further studied.

## Data availability statement

Publicly available datasets were analyzed in this study. This data can be found here: Gene Expression Omnibus (http://www.ncbi.nlm.nih.gov/geo) and GDC (https://portal.gdc.cancer.gov).

## Author contributions

YS, JP, and LH participated in the determination of research ideas and writing manuscripts. ZF was responsible for *in vitro* experimental verification. YS and KH conducted bioinformatics analysis and the results were visualized. TL, TC, and PZ participated in the writing and revision of manuscripts. All authors contributed to the article and approved the submitted version.

## Funding

The research project is supported by the National Natural Science Foundation of China (grant nos. 82060492), the National College Students Innovation and Entrepreneurship Training Program (202110403003), and the Jiangxi Provincial Science Fund for Distinguished Young Scholars (20212ACB216011).

## Acknowledgments

We sincerely acknowledge the contributions of Sangerbox, a free online platform for data analysis (http://vip.sangerbox.com/).

## Conflict of interest

The authors declare that the research was conducted in the absence of any commercial or financial relationships that could be construed as a potential conflict of interest.

## Publisher’s note

All claims expressed in this article are solely those of the authors and do not necessarily represent those of their affiliated organizations, or those of the publisher, the editors and the reviewers. Any product that may be evaluated in this article, or claim that may be made by its manufacturer, is not guaranteed or endorsed by the publisher.
