# Supplementary figures and images for: Osteosarcoma subtypes based on platelet-related genes and tumor microenvironment characteristics
Source: Front Oncol. 2022 Sep 23;12:941724. doi: 10.3389/fonc.2022.941724 (PMC9539847; doi:10.3389/fonc.2022.941724)

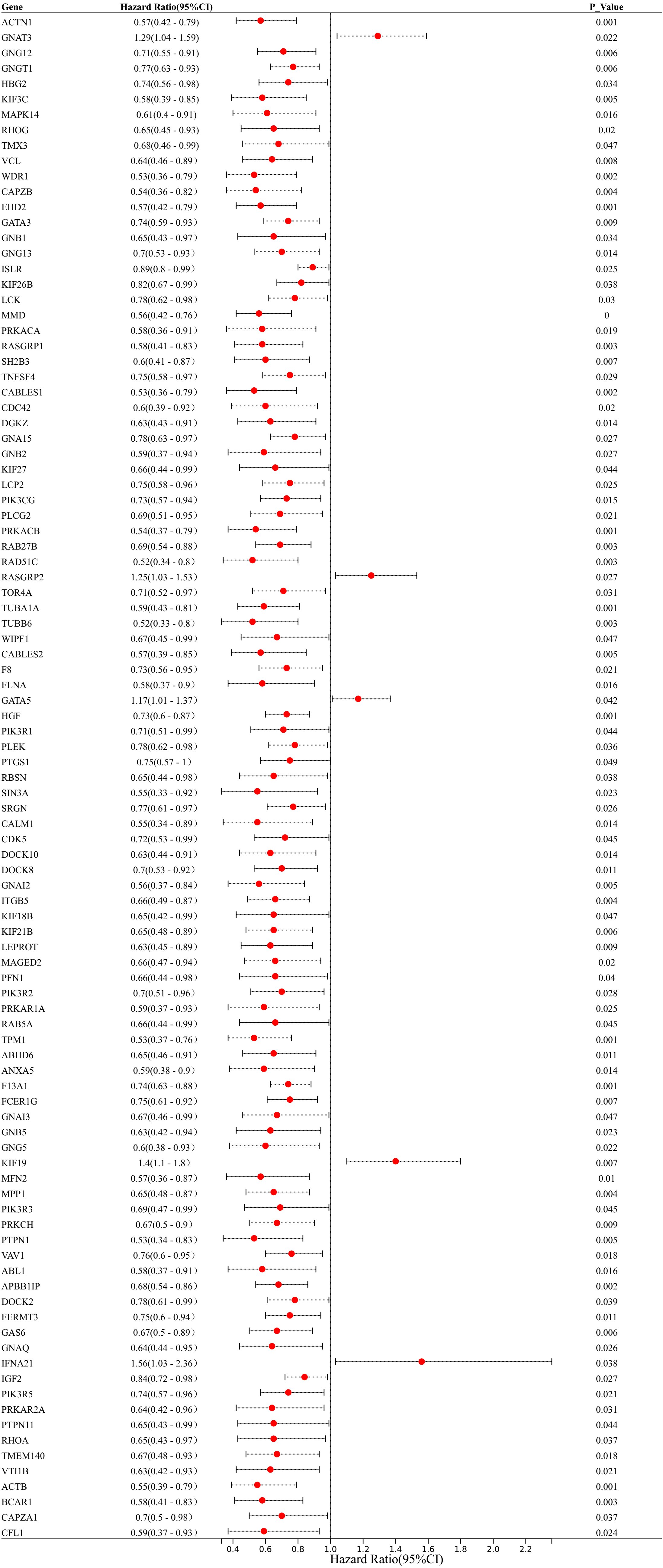

Supplement: Supplementary file 1 [file DataSheet_1.pdf]
